# Microarray data integration for genome-wide analysis of human tissue-selective gene expression

**DOI:** 10.1186/1471-2164-11-S2-S15

**Published:** 2010-11-02

**Authors:** Liangjiang Wang, Anand K Srivastava, Charles E Schwartz

**Affiliations:** 1 Department of Genetics and Biochemistry, Clemson University, Clemson, SC 29634, USA; 2 J.C. Self Research Institute of Human Genetics, Greenwood Genetic Center, Greenwood, SC 29646, USA

## Abstract

**Background:**

Microarray gene expression data are accumulating in public databases. The expression profiles contain valuable information for understanding human gene expression patterns. However, the effective use of public microarray data requires integrating the expression profiles from heterogeneous sources.

**Results:**

In this study, we have compiled a compendium of microarray expression profiles of various human tissue samples. The microarray raw data generated in different research laboratories have been obtained and combined into a single dataset after data normalization and transformation. To demonstrate the usefulness of the integrated microarray data for studying human gene expression patterns, we have analyzed the dataset to identify potential tissue-selective genes. A new method has been proposed for genome-wide identification of tissue-selective gene targets using both microarray intensity values and detection calls. The candidate genes for brain, liver and testis-selective expression have been examined, and the results suggest that our approach can select some interesting gene targets for further experimental studies.

**Conclusion:**

A computational approach has been developed in this study for combining microarray expression profiles from heterogeneous sources. The integrated microarray data can be used to investigate tissue-selective expression patterns of human genes.

## Background

There are many different types of cells in the human body, and similar cells group together to form a tissue with a specialized function. Multiple tissues constitute an organ such as brain, heart or liver. Gene expression variation is the primary determinant of tissue identity and function. Certain genes are expressed specifically or preferentially in a particular tissue. These genes are broadly called tissue-selective genes [1]. Note that tissue specificity is regarded as a special case of tissue selectivity, and tissue-specific genes are expressed only in a particular tissue. It is a fundamental question in biology to understand how selective gene expression underlies tissue development and function. Moreover, tissue-selective genes are implicated in many complex human diseases [2], and identification of these genes may provide valuable information for developing novel biomarkers and drug targets [1].

Tissue-selective expression was traditionally studied at the single-gene level with time-consuming techniques such as Northern blot and *in situ* hybridization. With the recent development of high-throughput technologies, biologists can perform genome-wide gene expression profiling in various tissues. These high-throughput technologies include Expressed Sequence Tag (EST) sequencing, Serial Analysis of Gene Expression (SAGE), and DNA microarrays. Yu et al. [3] analyzed the NCBI EST database (dbEST) to select a set of human genes that are preferentially expressed in a tissue of interest. The selection was based on the expression enrichment score, which was defined as the ratio between observed and expected number of ESTs for a gene. For the selected tissue-selective genes, regulatory modules were detected by examining the promoter motifs and their relationships with transcription factors. However, EST data are generated mainly for transcript sequence information, and EST counts can only be used as rough estimates of gene expression levels. Siu et al. [4] investigated gene expression patterns in different regions of the human brain by using SAGE, and identified some brain region-selective genes. Kouadjo et al. [5] also used the SAGE strategy to identify housekeeping and tissue-selective genes in fifteen mouse tissues. While SAGE tag counts can provide reliable estimation of gene expression, it is rather inefficient and expensive to use SAGE for profiling a large number of tissue samples with biological replicates.

The DNA microarray technology has been widely used to simultaneously profile the levels of thousands of mRNA transcripts in various tissues, and may hold great promise for elucidating the molecular mechanisms of complex human diseases. Many microarray datasets have been generated for identifying disease-associated biomarkers, classifying disease types, and predicting treatment outcomes. However, only a few microarray studies were designed to investigate human tissue-selective gene expression. Su et al. [6] used custom oligonucleotide arrays to examine the expression patterns of predicted genes across a panel of human and mouse tissues. The NCBI Gene Expression Omnibus (GEO at http://www.ncbi.nlm.nih.gov/geo/) has an Affymetrix microarray dataset for human body index of gene expression (GEO accession: GSE7307). Since each individual dataset does not contain a large number of expression profiles of various tissues, computational methods may be used to integrate the gene expression data from different microarray studies. Greco et al. [7] investigated tissue-selective expression patterns with an integrated dataset of microarray profiles publicly available at the GEO database. The relatively small dataset contained 195 expression profiles from six different microarray studies. The results suggested that gene expression data from Affymetrix GeneChip experiments could be integrated through pre-processing raw data (CEL files) with commonly used methods.

In this study, we have compiled a compendium of 2,968 microarray expression profiles of various human tissues from the NCBI GEO database. These expression profiles have been selected from 131 microarray datasets generated at different laboratories. Our data integration approach includes microarray data normalization, transformation, and quality control. The integrated data have been used to identify brain, liver and testis-selective genes using a new computational method based on both microarray hybridization intensities and detection calls. The results further suggest that the publicly available microarray expression profiles from heterogeneous sources can be integrated into a single dataset for examining gene expression patterns across various tissues.

## Methods

### Collection and curation of microarray gene expression profiles

Human microarray gene expression data are accumulating in public databases. These expression profiles have been generated for various research objectives, and show significant variations in data quality. To compile a compendium of high-quality microarray profiles for studying gene expression patterns, we manually curated the human microarray data publicly available in the NCBI GEO database (as of November 3, 2009). The following criteria were used to select microarray expression profiles in this study. First, the profiles had to be generated using the Affymetrix HG-U133 Plus 2.0 Array, a platform for complete coverage of the human genome with 54,675 probe sets. This array platform was used by the majority of human gene expression profiles deposited in the GEO database. Second, a detailed description of the microarray profiling study and raw data in CEL file format was available. The description contained important information about a microarray sample (e.g., tissue source, clinical condition, treatment, etc). Third, the expression profiles had to be obtained using normal tissue samples. Microarray profiles of cancer cells or diseased tissues were excluded from selection. Fourth, the tissue sample used for microarray profiling should not be cultured *in vitro* or treated with any drugs before RNA extraction. No expression profiles of primary or secondary cell cultures were selected for this study.

By following the above criteria, we compiled 3,030 microarray gene expression profiles across a variety of human tissues (Table 1). The number of selected profiles varied among tissues, depending on data availability. An attempt was made to include as many tissues as possible, even though some tissues had only a few expression profiles available in the GEO database. Nevertheless, some tissues had a relatively large number of expression profiles, and were thus particularly suited for identifying tissue-selective genes. For instance, there were 645 brain gene expression profiles (616 profiles after data quality control). These expression profiles were obtained from various regions of postmortem brain such as entorhinal cortex, hippocampus and cerebellum, and could be used to identify genes specifically expressed in neurons.

**Table 1:** List of human tissues and microarray expression profiles

Tissue	# of profiles selected	# of profiles integrated
Brain (various regions)	645	616
Pituitary gland	12	12
Thyroid gland	16	9
Adrenal gland	25	25
Pancreas	56	55
Skeletal muscle	122	109
Skin	101	101
Adipose tissue	80	80
Retina	12	12
Gingiva	71	71
Salivary gland	18	18
Tongue	22	20
Stomach	51	51
Small intestine	59	59
Colon	107	105
Liver	117	117
Kidney	73	73
Breast	132	132
Ovary	61	59
Uterus	117	117
Placenta	56	56
Umbilical cord	54	54
Testis	36	36
Prostate	58	58
Nasal epithelium	31	31
Airway epithelium	89	89
Lung	66	66
Alveolar macrophage	88	87
Heart	31	31
Tonsil	13	13
Lymph node	14	14
Blood (various cell types)	413	409
Other tissues	184	183

### Microarray data normalization and integration

Microarray raw data in CEL file format were downloaded from the GEO database, and then normalized by using the dChip software (available at http://www.dchip.org). As a widely used tool for microarray data analysis, dChip can display and normalize CEL files with a model-based approach [8]. For a given group of CEL files, dChip can be used to calculate the model-based expression values and make the qualitative detection calls for each array. The detection call (Present, Marginal or Absent) provides a statistical assessment about whether the perfect matches (PMs) show significantly more hybridization signal than the corresponding mismatches (MMs) in a probe set. Since the detection call and expression level are computed in different ways, a gene transcript with an Absent call may still be given an expression value (although usually low).

**Figure 1:**
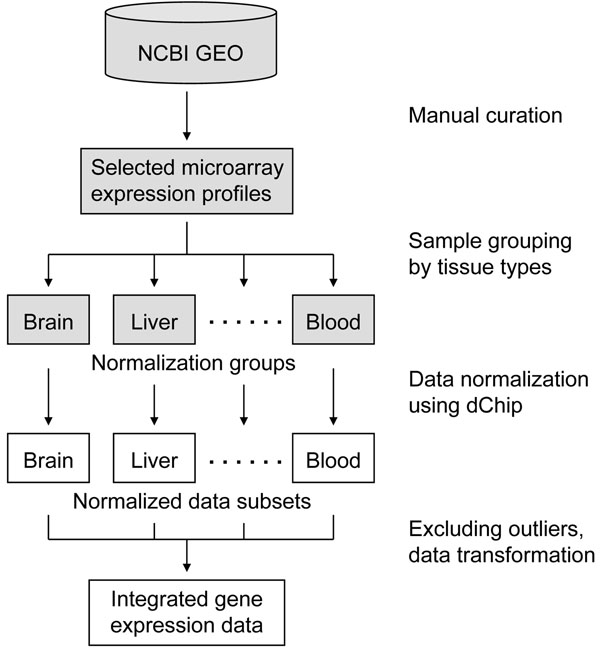
Schematic diagram of microarray data normalization and integration.

One challenging task in this study was to combine the expression profiles of various tissue types and from different microarray studies into a single integrated dataset. As outlined in Figure 1, our approach included the following steps. First, the selected microarray CEL files were organized into different normalization groups, each of which contained expression profiles of the same or similar tissue type. For example, one normalization group was consisted of 117 liver microarray profiles, whereas another group contained 112 expression profiles of six endocrine glands, including pituitary gland (12 profiles), thyroid gland (16 profiles), parathyroid gland (1 profile), thymus gland (2 profiles), adrenal gland (25 profiles) and pancreas (56 profiles). Within a normalization group, the variation of tissue type was thus minimized although the expression profiles were nevertheless obtained from different microarray studies.

Second, each group of microarray profiles was normalized by using the invariant set method [9]. For each normalization group, the expression profile with median overall intensity was chosen as the baseline array, against which the other profiles were normalized at probe intensity level. A subset of PM probes with small rank difference between the profile to be normalized and the baseline array were chosen as the invariant set for fitting a normalization curve. The normalization transformation was then performed for all the probes in the profile based on the curve [9]. While the invariant set normalization method could reduce the variation in microarray profiles from different studies, it might not be applied to an expression dataset with various tissue types. Owing to the biological variation of gene expression across different tissues, a baseline array should be used to normalize the microarray profiles of each tissue type (or similar tissues).

Finally, the normalized microarray profiles were integrated into a single dataset after outlier array exclusion and global median transformation. When fitting the statistical model to a probe set, dChip used an outlier detection algorithm to identify array-outliers whose response pattern for the probe set was significantly different from the consensus probe response pattern in the other arrays [8]. After the model was fitted for all probe sets, the percentage of probe sets detected as array-outliers was calculated for each array. If the percentage exceeded 15%, the array was discarded as an outlier array. In this study, only 62 outlier arrays were detected for all the 3,030 selected expression profiles (Table 1). Global median transformation was then applied to the remaining profiles. Each expression value in a profile was divided by the profile’s median value. The transformation was necessary because the expression profiles from different normalization groups often had different median values. Thus, the integrated dataset had 2,968 expression profiles with the same median value (i.e., 1.00).

### Genome-wide identification of tissue-selective genes

In this study, a new computational method has been designed to analyze the integrated microarray data for identifying tissue-selective genes, which refers to the genes specifically or preferentially expressed in a particular tissue. The computational task is not trivial for the following reasons. First, the expression profiles have been compiled from various studies, in which tissues at different ages and in different conditions were used for microarray profiling. Thus, the microarray profiles of the same tissue type should not be considered as biological replicates. Second, some tissue-selective genes can be expressed at certain developmental stages or in specific conditions, and their expression may not be consistently detected in all the microarray profiles of a tissue type. Third, microarray data are inherently noisy. It was thus desired that both the expression values and detection calls of microarray profiles can be utilized for tissue-selective gene identification.

**Figure 2:**
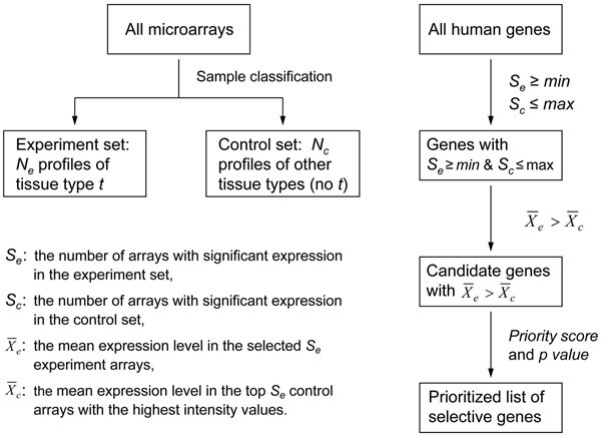
Schematic diagram of microarray data analysis for genome-wide identification of tissue-selective genes.

Figure 2 illustrates our approach for genome-wide identification of tissue-selective genes. First, for a given tissue type *t*, the microarray expression profiles are divided into two sets: experiment set and control set. The experiment set contains the expression profiles of tissue type *t*, and the control set has the expression profiles of the other tissue types. The experiment set usually has fewer microarray profiles than the control set. For example, to identify brain-selective genes in this study, the experiment set contained 616 expression profiles, whereas the control set had 2,352 expression profiles of the other tissue types such as liver, kidney, muscle, skin, etc.

Second, all the human genes (array probe sets) are examined for significant expression in the microarray profiles. The term “significant expression” in this study is used to describe gene expression data that meet the following two criteria: (1) the detection call is Present; and (2) the expression value is no less than a threshold *θ* (*θ* ≥ 0). Since there are no negative values in a microarray profile, significant expression would be solely defined by the detection call if *θ* = 0. For each probe set, the number of significant expression in the experiment set (*S_e_*) and that in the control set (*S_c_*) are calculated. Genes that have *S_e_* ≥ *min* and *S_c_* ≤ *max* are selected for further analyses. The threshold *min* is used to specify the minimum number of significant expression that should be detected in the experiment set. Considering the noise in microarray data, significant expression may also be detected in the control set, but the number *S_c_* should not exceed *max* (maximum number of significant expression). The threshold *max* is set to 0 if no observation of significant expression is allowed in the control set. For a tissue-selective gene, its frequency of significant expression should be higher in the experiment set than in the control set. *Score*1 is calculated as follows:

 (1)

where *N_e_* is the total number of expression profiles in the experiment set, and *N_c_* is the total number of expression profiles in the control set.

Third, for each selected probe set, its expression level in the experiment set is compared with that in the control set. Our assumption is that potential tissue-selective genes should show higher expression in the experiment arrays than in the control arrays. *Score*2 is calculated as follows:

 (2)

where  is the mean expression level of the selected probe set in the *S_e_* experiment arrays with significant expression, and  is the mean expression level in control arrays. In this study, the control arrays were sorted according to their expression values for the selected probe set, and the top *S_e_* control arrays with the highest expression values were used to compute the mean, . The probe sets with *Score*2 ≤ 0 were excluded from consideration for tissue-selective genes.

Finally, the potential tissue-selective gene targets are prioritized according to the overall score, which is calculated as follows:

 (3)

where *w*_1_ and *w*_2_ are two weights for *Score*1 and *Score*2, respectively. In this study, *w*_1_ = 1 and *w*_2_ = 1 were used to calculate the priority score for each selected probe set. Moreover, the statistical significance of the tissue-selective expression pattern was evaluated by the permutation analysis. The hybridization signals of a probe set, including its expression values and detection calls, were permuted, and then divided into the experiment and control set to calculate the priority score. After one million permutations were performed for each selected probe set, the significance level (*p*-value) was calculated as the fraction of permutations that gave rise to scores greater than or equal to the actual priority score of the probe set. The *p*-value thus provided an estimation of the probability for observing the tissue-selective expression pattern by chance.

## Results and discussion

A compendium of 2,968 expression profiles of various human tissues have been compiled from 131 microarray studies. These expression profiles have been combined into a single dataset after global normalization, and then used for the genome-wide analysis of tissue-selective gene expression. Although the analysis can be performed for any tissues with available microarray data (Table 1), we present in this paper the results from three case studies on brain, liver and testis-selective gene expression.

### Brain-selective gene expression

The human brain is highly complex, and contains 50-100 billion neurons. There are many different brain regions with specific functions. For example, the frontal lobe is involved in higher mental functions and long-term memories, whereas the occipital lobe is the visual processing center. In this study, the microarray expression profiles of different brain regions were combined into the experiment set (616 profiles), and compared with the expression profiles of non-brain tissues in the control set (2,352 profiles). Thus, the brain-selective genes identified in this study might be involved in basic neuron functions such as neural signal processing and transmission via synapses (complex membrane junctions between neurons).

**Figure 3:**
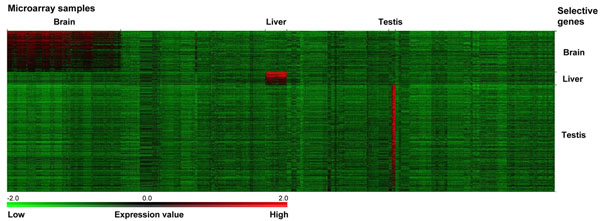
Visualization of tissue-selective gene expression patterns. The normalized expression values were transformed by logarithm (base 10), and each microarray profile had the median value of zero.

Table 2 shows the top 20 high-scoring genes from one of the analyses with different parameter settings. In this analysis, significant expression was defined by the detection call being Present and the relative expression value no less than 1.00 (array median value). The minimum number of significant expression in the experiment group (*min*) was set to 62 (~10% of experiment arrays), and the maximum number of significant expression in the control group (*max*) was set to 24 (~1% of control arrays). With the above parameters, 222 genes have been identified as brain-selective targets with the priority score ranging from 1.18 to 4.69 (see Additional file 1). The permutation analysis suggests that the brain-selective expression patterns of all the selected genes are statistically significant (*p* < 0.000001). In Figure 3, the gene expression patterns are visualized with the heat map generated by using TM4 MeV [10]. Clearly, the transcripts of the selected genes are predominantly detected in brain samples.

**Table 2:** List of high-scoring genes with selective expression in the brain ^1^

Probe	Gene	Description	*S_e_*	*S_c_*		Score
205914_s	*GRIN1*	Glutamate receptor, ionotropic, N-methyl D-aspartate 1	284	0	4.54	4.69
236324	*MBP*	Myelin basic protein	211	0	5.13	4.62
238061	*LGI3*	Leucine-rich repeat LGI family gene 3	536	1	14.77	4.48
205989_s	*MOG*	Myelin oligodendrocyte glycoprotein	569	2	24.03	4.42
206899	*NTSR2*	Neurotensin receptor 2	380	1	16.49	4.38
203540	*GFAP*	Glial fibrillary acidic protein	604	14	115.10	4.28
244113	-	cDNA sequence (GB: R44603)	595	3	24.55	4.27
231489_x	-	cDNA sequence (GB: H12214)	551	18	94.24	4.04
206970	*CNTN2*	Contactin 2 (axonal)	407	1	6.67	4.02
224536_s	*PCDHGC5*	Protocadherin gamma subfamily C, 5	68	0	3.37	3.94
1556877	-	cDNA sequence (GB: BC040662)	364	1	5.48	3.88
235375_x	*TTC9B*	Tetratricopeptide repeat domain 9B	408	3	13.64	3.85
208320	*CABP1*	Calcium binding protein 1	365	5	18.23	3.71
230255	*GABRD*	GABA-A receptor subunit delta	451	8	23.42	3.70
235794	*MOBP*	Myelin-associated oligodendrocyte basic protein	596	12	25.64	3.69
206678	*GABRA1*	GABA-A receptor subunit alpha 1	532	15	35.05	3.68
233471	*PTPN5*	Protein tyrosine phosphatase, non-receptor type 5	350	2	5.52	3.57
1557481_a	*C21orf131*	Chromosome 21 open reading frame 131	474	3	5.92	3.55
219642_s	*PEX5L*	Peroxisomal biogenesis factor 5-like	415	6	12.91	3.53
232409_x	*FBXL16*	F-box and leucine-rich repeat protein 16	227	3	11.70	3.53

^1^ The number of significant expression in the experiment set (*S_e_*) and that in the control set (*S_c_*) are shown together with the ratio of the mean expression level in the experiment arrays to the level in the control arrays ().

**Table 3:** List of high-scoring genes with selective expression in the liver **^1^**

Probe	Gene	Description	*S_e_*	*S_c_*		Score
210798_x	*MASP2*	Mannan-binding lectin serine peptidase 2	116	0	26.61	5.88
208088_s	*CFHR5*	Complement factor H-related 5	110	1	95.56	5.41
1554459_s	*CFHR3*	Complement factor H-related 3	116	5	103.31	4.77
37020	*CRP*	C-reactive protein, pentraxin-related	110	18	228.26	4.53
210049	*SERPINC1*	Serpin peptidase inhibitor, clade C, 1	117	18	213.02	4.53
205754	*F2*	Coagulation factor II (thrombin)	117	13	135.57	4.47
207406	*CYP7A1*	Cytochrome P450, 7A1	56	1	19.52	4.43
207097_s	*SLC17A2*	Solute carrier family 17, member 2	117	6	45.67	4.34
207874_s	*CFHR4*	Complement factor H-related 4	115	18	138.54	4.33
207027	*HGFAC*	HGF activator	112	4	22.37	4.18
220224	*HAO1*	Hydroxyacid oxidase 1	117	18	86.38	4.14
231703_s	*ADH4*	Alcohol dehydrogenase 4 (class II), pi	89	3	18.26	4.12
224243	*APOA5*	Apolipoprotein A-V	109	3	14.26	4.10
207256	*MBL2*	Mannose-binding lectin 2, soluble	117	18	74.97	4.07
219903_s	*CYP2C8*	Cytochrome P450, 2C8	61	1	7.03	4.02
1557226_a	*ASPG*	Similar to asparaginase	95	2	8.27	3.98
231702	*TDO2*	Tryptophan 2,3-dioxygenase	84	3	13.10	3.95
231662	*ARG1*	Arginase, liver	102	14	40.21	3.85
210326	*AGXT*	Alanine-glyoxylate aminotransferase	117	8	14.95	3.73
237765	*C14orf68*	Chromosome 14 open reading frame 68	117	13	23.52	3.71

^1^ The number of significant expression in the experiment set (*S_e_*) and that in the control set (*S_c_*) are shown together with the ratio of the mean expression level in the experiment arrays to the level in the control arrays ().

Perhaps more importantly, many genes identified in this study have been previously suggested to be expressed specifically or preferentially in the brain. These genes include *GRIN1, MBP, LGI3, MOG, NTSR2, GFAP, CNTN2, PCDHGC5, CABP1, GABRD, MOBP* and *GABRA1* (Table 2). The protein encoded by the *GRIN1* gene is a critical subunit of the glutamate receptor channel, and plays a key role in the plasticity of synapses underlying memory and learning [11]. Genetic alterations in *GRIN1* have been shown to be associated with Alzheimer's disease [12] and bipolar disorder [13]. In this study, *GRIN1* has the highest priority score with significant expression in 284 brain samples but none in the other tissues (Table 2). *GABRD* and *GABRA1* encode two subunits of the GABA-A receptor, which binds the major inhibitory neurotransmitter GABA in the brain [14]. GABA-A receptors are chloride channels that regulate membrane potential, and play structural roles in synapse maturation and stabilization. *LGI3* encodes a leucine-rich repeat protein involved in the regulation of neuronal exocytosis [15]. *CABP1* is a neuron-specific member of the calmodulin superfamily, and modulates Ca^2+^-dependent activity of inositol 1, 4, 5-trisphosphate receptors [16]. Both *CNTN2* and *PCDHGC5* encode immunoglobulin-like proteins important for the establishment and function of neural connections in the brain [17,18]. In addition, *MBP, MOG* and *MOBP* encode constituents of the myelin sheath of oligodendrocytes, and *GFAP* encodes an intermediate filament protein of mature astrocytes in the central nervous system.

However, the expression and function of many other genes selected by the above analysis have not been well documented in the literature. For example, the TTC9B protein contains the tetratricopeptide repeat domain, and is conserved in other mammals, but its function in the brain is still unclear. In this study, the *TTC9B* gene shows significant expression in 408 out of 616 brain samples (Table 2). By contrast, in only 3 out of 2,352 control samples, significant expression is detected. Moreover, the mean expression level of *TTC9B* in the brain samples is 13.64-fold higher than that in the other tissues. As shown in Table 2, brain-selective expression patterns have also been demonstrated for four other genes (*PTPN5*, *C21orf131*, *PEX5L* and *FBXL16*) and three cDNA sequences (R44603, H12214 and BC040662), even though their functions in the brain remain to be characterized. The three sequences were obtained from brain cDNA libraries, but their corresponding genes were not determined. Altogether, the results suggest that the approach developed in this study can be used to not only confirm the brain-selective expression of some known genes, but also identify interesting targets for further experimental studies.

### Liver-selective gene expression

The liver plays a key role in metabolism, and its functions include plasma protein synthesis, detoxification, and production of bile necessary for digestion. To identify liver-selective genes, the microarray data were grouped into the experiment set consisting of 117 liver expression profiles and the control set containing 2,851 profiles of non-liver tissues. The parameters for the analysis are as follows: *θ* = 1.00 (array median value), *min* = 23 (~20% of liver arrays), and *max* = 29 (~1% of control arrays), where *θ* is the relative intensity threshold for significant expression, *min* is the minimum number of significant expression in the experiment set, and *max* is the maximum number of significant expression in the control set. There are 69 gene targets identified for potential liver-selective expression, and the priority score ranges from 1.64 to 5.88 (see Additional file 2). Based on the permutation analysis, the liver-selective expression patterns of all the selected genes are statistically significant (*p* < 0.000001). The expression patterns of these genes are shown in Figure 3.

Interestingly, 17 of the top 20 high-scoring genes listed in Table 3 are previously known to be expressed predominantly in the liver. In particular, nine genes (*MASP2, CFHR5, CFHR3, CRP, SERPINC1, F2, CFHR4, APOA*5 and *MBL2*) are highly expressed in the liver, and their protein products are secreted to blood plasma. *MASP2, CFHR5, CFHR3, CRP, CFHR4* and *MBL2* play important roles in the innate immune defense against pathogens [19]. *SERPINC1* and *F2* are involved in regulating the blood coagulation cascade [20]. *APOA5* encodes an apolipoprotein important for the regulation of plasma triglyceride level, a major risk factor for coronary artery disease [21]. Six of the known liver-selective genes encode metabolic enzymes involved in cholesterol catabolism and bile acid biosynthesis (*CYP7A1*), the urea cycle (*ARG1*), glyoxylate detoxification (*AGXT*), and the oxidation of alcohols (*ADH4*) and other compounds (*CYP2C8* and *HAO1*). In addition, *HGFAC* encodes a peptidase involved in hepatocyte growth factor activation, and *C14orf68* encodes a liver-specific mitochondrial carrier protein. The other three high-scoring genes (*SLC17A2*, *ASPG* and *TDO2*) have not been previously shown to be expressed preferentially in the liver.

### Testis-selective gene expression

When compared with brain and liver tissues, many other tissues have fewer number of microarray expression profiles available (Table 1). The microarray dataset has only 36 expression profiles of the testis, which produces sperm and male sex hormones. To identify testis-selective genes, these 36 expression profiles (experiment set) were compared with 2,932 microarray profiles of non-testis tissues (control set) by using the following parameters: *θ* = 1.00 (array median value), *min* = 7 (~20% of testis arrays), and *max* = 29 (~1% of control arrays). The analysis resulted in 581 gene targets with the priority score ranging from 1.35 to 6.05 (see Additional file 3). The testis-selective expression patterns of these targets were found to be statistically significant by permutation testing (*p* < 0.000001). Figure 3 shows the expression patterns of the testis-selective gene targets.

As listed in Table 4, the top 20 high-scoring targets include five known testis-selective genes (*C9orf11, TNP2, TSSK3, ACTL7B* and *NT5C1B*). The *C9orf11* gene encodes a vesicle membrane protein involved in the biogenesis of acrosome, a cap-like structure that covers the anterior half of the head in the spermatozoa [22]. *TNP2* encodes a chromosomal transition protein for the conversion of nucleosomal chromatin to the compact form found in the sperm nucleus [23]. *TSSK3* encodes a protein kinase expressed exclusively in the testis, and may be involved in signal transduction during male germ cell development or mature sperm function [24]. *ACTL7B* and *NT5C1B* are expressed preferentially in the testis, but their exact functions are still unknown.

**Table 4:** List of high-scoring genes with selective expression in the testis ^1^

Probe	Gene	Description	*S_e_*	*S_c_*		Score
1554981	*C9orf11*	Chromosome 9 open reading frame 11	19	0	71.87	6.05
207736_s	*TNP2*	Transition protein 2 (histone to protamine)	18	0	52.45	5.89
231563	-	cDNA sequence (GB: BF508261)	19	0	49.40	5.88
237319	*C2orf53*	Chromosome 2 open reading frame 53	14	0	36.33	5.62
1552395	*TSSK3*	Testis-specific serine kinase 3	17	0	22.10	5.49
1562864	-	cDNA sequence (GB: BC033504)	15	0	22.90	5.45
243494	-	cDNA sequence (GB: AI204633)	19	0	15.59	5.38
243143	*FAM24A*	Family with sequence similarity 24, A	18	0	15.27	5.35
231362	-	cDNA sequence (GB: AI423933)	19	0	14.14	5.34
1560494_a	*CPXCR1*	CPX chromosome region, candidate 1	17	0	14.38	5.30
236661	*IQCF6*	IQ motif containing F6	18	0	12.53	5.26
220498	*ACTL7B*	Actin-like 7B	18	0	11.49	5.23
1556740	-	cDNA sequence (GB: AA398245)	12	0	16.70	5.21
1554368	*NT5C1B*	5'-nucleotidase, cytosolic IB	19	1	99.85	5.19
241527	-	cDNA sequence (GB: AI799028)	16	0	11.57	5.18
1561704	-	cDNA sequence (GB: BC041892)	11	0	16.40	5.17
241518	-	cDNA sequence (GB: AA428659)	11	0	16.11	5.16
242925	*RNF148*	Ring finger protein 148	19	1	91.88	5.15
1554855	*PARK2*	E3 ubiquitin-protein ligase parkin	10	0	16.74	5.13
1556207_a	-	cDNA sequence (GB: BC035261)	17	0	8.65	5.08

^1^ The number of significant expression in the experiment set (*S_e_*) and that in the control set (*S_c_*) are shown together with the ratio of the mean expression level in the experiment arrays to the level in the control arrays ().

The other high-scoring targets have not been previously shown to be testis-selective genes. *PARK2* is known to be expressed in the brain, and mutations in this gene cause Parkinson disease [25]. The results from this study suggest that the highest expression of *PARK2* appears to occur in the testis (Table 4). There are five other genes (*C2orf53, FAM24A, CPXCR1, IQCF6* and *RNF148*) whose expression and function in the testis have not been well documented in the literature. In addition, the high-scoring targets include nine cDNA sequences. Interestingly, all the sequences except BC033504 and AI423933 were obtained from testis cDNA libraries (BC033504 from a brain library and AI423933 from a glioblastoma library). Considering the relative small sample size of testis expression profiles, it is uncertain whether all the selected probe sets represent true testis-selective genes. However, the targets with high priority scores should provide a good starting point for experimental studies on testis-selective gene expression and function.

## Conclusion

A comprehensive microarray dataset has been compiled in this study for genome-wide analysis of human tissue-selective gene expression. The dataset contains 2,968 expression profiles of various normal tissues from 131 microarray studies. A new computational method has been designed to identify tissue-selective genes using both microarray intensity values and detection calls. To demonstrate that the integrated microarray data can be used to investigate human gene expression patterns, we have examined the lists of potential brain, liver and testis-selective genes. Notably, many of the high-scoring targets are actually known tissue-selective genes, suggesting that the approach developed in this study works effectively. Furthermore, the approach can be used to identify some interesting targets with tissue-selective expression patterns. These targets may be used for further experimental studies on human gene expression and function.

## Competing interests

The authors declare that they have no competing interests.

## Authors' contributions

LW conceived and designed the study, conducted the data analysis, and drafted the manuscript. AKS and CES contributed to result interpretation and manuscript preparation.

## Supplementary Material

Additional file 1 List of brain-selective gene targets
**List of brain-selective gene targets.** The full list of potential brain-selective genes identified in this study. Click here for file

Additional file 2 **List of liver-selective gene targets.** The full list of potential liver-selective genes identified in this study. Click here for file

Additional file 3 **List of testis-selective gene targets.** The full list of potential testis-selective genes identified in this study. Click here for file
